# Nephroprotective Effects of Synthetic Flavonoid Hidrosmin in Experimental Diabetic Nephropathy

**DOI:** 10.3390/antiox10121920

**Published:** 2021-11-29

**Authors:** Luna Jiménez-Castilla, Gema Marín-Royo, Macarena Orejudo, Lucas Opazo-Ríos, Teresa Caro-Ordieres, Inés Artaiz, Tatiana Suárez-Cortés, Arturo Zazpe, Gonzalo Hernández, Carmen Gómez-Guerrero, Jesús Egido

**Affiliations:** 1 Renal, Vascular and Diabetes Research Laboratory, IIS-Fundación Jiménez Díaz, Universidad Autónoma de Madrid, 28040 Madrid, Spain; luna.jimenez@quironsalud.es (L.J.-C.); gmarinroyo@gmail.com (G.M.-R.); macaorejudo@gmail.com (M.O.); JEgido@quironsalud.es (J.E.); 2 Spanish Biomedical Research Centre in Diabetes and Associated Metabolic Disorders (CIBERDEM), 28029 Madrid, Spain; 3 Department of Research, Development, and Innovation, FAES Farma, 48940 Leioa, Spain; tcaro@faes.es (T.C.-O.); iartaiz@faes.es (I.A.); tsuarez@faes.es (T.S.-C.); AZazpe@faes.es (A.Z.); ghernandez@faes.es (G.H.)

**Keywords:** hidrosmin, diabetic nephropathy, albuminuria, inflammation, oxidative stress

## Abstract

Diabetes mellitus (DM) is a high-impact disease commonly characterized by hyperglycemia, inflammation, and oxidative stress. Diabetic nephropathy (DN) is a common diabetic microvascular complication and the leading cause of chronic kidney disease worldwide. This study investigates the protective effects of the synthetic flavonoid hidrosmin (5-O-(beta-hydroxyethyl) diosmin) in experimental DN induced by streptozotocin injection in apolipoprotein E deficient mice. Oral administration of hidrosmin (300 mg/kg/day, *n* = 11) to diabetic mice for 7 weeks markedly reduced albuminuria (albumin-to-creatinine ratio: 47 ± 11% vs. control) and ameliorated renal pathological damage and expression of kidney injury markers. Kidneys of hidrosmin-treated mice exhibited lower content of macrophages and T cells, reduced expression of cytokines and chemokines, and attenuated inflammatory signaling pathways. Hidrosmin treatment improved the redox balance by reducing prooxidant enzymes and enhancing antioxidant genes, and also decreased senescence markers in diabetic kidneys. In vitro, hidrosmin dose-dependently reduced the expression of inflammatory and oxidative genes in tubuloepithelial cells exposed to either high-glucose or cytokines, with no evidence of cytotoxicity at effective concentrations. In conclusion, the synthetic flavonoid hidrosmin exerts a beneficial effect against DN by reducing inflammation, oxidative stress, and senescence pathways. Hidrosmin could have a potential role as a coadjutant therapy for the chronic complications of DM.

## 1. Introduction

Diabetes mellitus (DM) is a chronic disease that appears when the pancreas is unable to produce enough insulin, or the body cannot use this hormone properly [1]. About 463 million people between 20 to 79 years have DM nowadays, and 10% of global health expenditure is estimated to be spent on this illness and its complications [2]. Uncontrolled DM in patients can result in prolonged hyperglycemia and the development of chronic complications classified in microvascular (retinopathy, nephropathy, and neuropathy)and macrovascular (coronary artery, peripheral, and cerebral vascular disease) [3].

Diabetic nephropathy (DN) is the main cause of end-stage chronic kidney disease globally and an important cardiovascular risk factor. The estimated prevalence of DN is approximately 30% and 50% in type 1 diabetes (T1D) and type 2 diabetes (T2D), respectively, and it is expected to increase despite the advances in DM management [4]. Diabetic kidney disease starts with microalbuminuria, defined as albumin excretion of 30–299 mg/day, and without treatment, it evolves into macroalbuminuria (>500 mg albumin/day) and progressive declining in the glomerular filtration rate [3]. The pathological situation in kidneys is characterized by increased glomerular basement membrane thickness, mesangial matrix expansion and nodule formation, tubulointerstitial inflammation, glomerulosclerosis, and interstitial fibrosis. [3,5].

At the molecular level, DN and its symptoms are the results of the addition of genetic factors, hyperglycemia, inflammation, oxidative stress, generation of advanced glycation end products, and overexpression of cytokines and growth factors. Particularly, inflammation has been studied as a focus in the DN development due to the presence of high concentrations of inflammatory cells both in glomeruli and tubulointerstitium of diabetic kidneys, as well as overexpression of chemokines, cytokines, and adhesion molecules [6].

Moreover, the chronic overactivation of proinflammatory pathways, such as nuclear factor-κB (NF-κB) and Janus kinase (JAK)/signal transducer and activator of transcription (STAT), is usual in the progression of DN. The activation of these processes triggers a cascade of the previously mentioned molecules [7]. Furthermore, the interaction between certain chemokines and their receptors leads to the recruitment of inflammatory cells into the organ involved, and proinflammatory cytokines contribute to the development and maintenance of the inflammation [6,8].

In addition to hyperglycemia and inflammation, oxidative stress also plays an important role, and they are very interconnected. DN affects the redox state by increasing reactive oxygen species (ROS) concentration and many molecules are affected in their conformational structures, producing disarrangements in the cellular functions [9]. In DN, redox imbalance occurs mainly due to the increase in ROS-producing enzymes, decrease in antioxidant enzymes, and transient induction of the NFE2-related factor 2 (NRF2) pathway as an antioxidant defense mechanism [10,11,12]. In addition to the changes in conformational structures of critical molecules of the cells, the overconcentration of ROS also leads to breaks in the DNA strains by disrupting the base (histones) or sugar moieties, which accelerates cellular senescence along with inflammation due to an augmentation of mutation rates and growth inhibition [12,13,14]. 

Strategies to treat DN have been mainly based on prevention by maintaining correct blood glucose control, cholesterol, and hypertension parameters attempting to reduce albuminuria and prevent the progression of renal failure. In the last few years, high-quality clinical trials have largely demonstrated that sodium-glucose cotransporter-2 inhibitors (SGLT2i) and glucagon-like peptide-receptor agonists (GLP-1 RA) offer cardiovascular and renal protection. In fact, both treatments have been included in the management recommendations in all DM clinical guidelines, mainly for patients with high cardiovascular and kidney risk [15,16,17]. However, in spite of the key role that inflammation and redox balance signaling pathways play in the genesis and progression of DN, no drug targeting those areas is available so far [7,18].

Recently, the use of flavonoids has emerged as a promising approach to treat DN. Flavonoids are plant secondary metabolites with a common C6-C3-C6 skeleton with two aromatic rings linked through a three-carbon bridge. These compounds are very usual, and they categorize according to their unsaturation degree and the substitution pattern [19,20]. 

Hidrosmin is a synthetic flavonoid developed for the treatment of venous insufficiency [21,22] (Supplementary Figure S1). Hidrosmin is derived from diosmin, a hesperidin-derivative bioflavonoid whose antioxidant, anti-inflammatory, antihypertensive, and anti-ischemic properties have been studied in several experimental models [23,24]. The aim of this study was to investigate the effect of hidrosmin on a well-established experimental model of DN and on cultured renal cells in order to identify its potential therapeutic actions as well as the underlying mechanisms.

## 2. Materials and Methods

### 2.1. Ethics Statement

All the in vivo experimental procedures carried out in this study were performed under the principle for replacement, refinement, or reduction (the 3Rs) and in accordance with the Directive 2010/63/EU of the European Parliament and were approved by the Institutional Animal Care and Use Committee of IIS-Fundación Jimenez Diaz and Community of Madrid (PROEX 116/16 and 217/19). Male Apolipoprotein E knockout (ApoE KO) mice (Jackson Laboratory, Bar Harbor, ME, USA) were housed in ventilated cages (2–3 mice per cage) with usual bedding material and environmental enrichment in a conventional temperature-controlled room (20–22 °C) with 12 h light/dark cycle and free access to water and standard food. 

### 2.2. Design of the Experimental DN Model

Experimental diabetes was induced in 14- to 16-week-old ApoE KO mice by intraperitoneal injection of streptozotocin (STZ, 125 mg/kg/day in 10 mmol/L citrate buffer, pH 4.5; S0130, Sigma-Aldrich, St. Louis, MO, USA) once a day for two consecutive days [25,26]. All mice were fed with a standard diet and water ad libitum during the experiments and were monitored every 2–3 days at the same time of the day for water consumption, body weight, and blood glucose (NovaPro glucometer, Nova Biomedical Iberia, Barcelona, Spain). After 2 weeks, mice with overt diabetes (glucose > 19.4 mmol/L) were randomized to receive orally through the feeding bottle vehicle (tap water; *n* = 9) or hidrosmin (5-O-(beta-hydroxyethyl) diosmin) at 300 mg/kg/day dissolved in tap water (*n* = 11) for 7 weeks. Both tap water and hidrosmin solution were renewed every 2–3 days. The dose of hidrosmin was decided according to safety and efficacy studies performed over the years by the pharmaceutical company that provided the drug for this study (FAES FARMA, Bilbao, Spain). At the end of the study, 12 h fasted mice were anesthetized (100 mg/kg ketamine and 15 mg/kg xylazine), saline perfused, and euthanized, and samples were collected. Dissected kidneys were snap frozen for RNA isolation or fixed in 10% formalin, paraffin embedded, and sectioned for histological analysis. Serum levels of glucose, urea, blood urea nitrogen, lipids, and transaminases were determined in a Roche Cobas autoanalyzer’s at the Central Laboratory of IIS-Fundación Jiménez Díaz (Madrid, Spain). Urinary creatinine concentrations were measured by colorimetric assay (ab65340, Abcam, Cambridge, UK) according to the manufacturer’s instructions. Albuminuria was determined by ELISA kit (ab108792, Abcam) and corrected for creatinuria to obtain urine albumin to creatinine ratio (UACR). Urine levels of kidney injury molecule-1 (KIM-1) were determined by ELISA kit (ab119596, Abcam).

### 2.3. Histological and Immunohistochemical Analysis

Paraffin kidney sections of 3 µm thickness were stained with periodic acid–Schiff (PAS) staining for histologic scoring of glomerular and tubulointerstitial damage. Renal lesions were semiquantitatively graded (0–3 scale) in a blinded manner according to the extent of glomerular changes (nodular sclerosis, mesangial matrix expansion, glomerulomegaly, and arteriolar hyalinosis) and tubulointerstitial lesions (tubular casts, acute tubular damage or flattening, tubular atrophy, and inflammatory infiltrate) [27,28]. Fibrosis was evaluated with Masson’s trichrome staining. All samples were evaluated by 20 fields per sample as previously described [26]. 

For immunohistochemistry, kidney tissue sections were deparaffinized and dehydrated through graded xylene and ethanol. After antigen retrieval (0.01 M citrate buffer pH 6 for 20 min) and blockade of endogenous peroxidase (3% H_2_O_2_ in methanol for 30 min) and nonspecific binding (8% host serum for 30 min), slides were incubated overnight at 4 °C with primary antibodies against CD3 (Agilent Cat# A0452, RRID:AB_2335677; Santa Clara, CA, USA), F4/80 (Bio-Rad Cat# MCA497R, RRID:AB_323279; Hercules, CA, USA), phosphorylated (p-)STAT3 (Cell Signaling Technology Cat# 9134, RRID:AB_331589; Danvers, MA, USA), p-p65 (Santa Cruz Biotechnology Cat# sc-136548, RRID:AB_10610391; Santa Cruz, CA, USA), p-NRF2 (Abcam Cat# ab76026, RRID:AB_1524049), p16^INK4a^ (Thermo Fisher Scientific Cat# MA5-17142, RRID:AB_2538613; Waltham, MA, USA), and p-H2A histone family member X (H2A.X)(Cell Signaling Technology Cat# 9718, RRID:AB_2118009). After rinsing in PBS, samples were incubated with biotinylated secondary antibodies, followed by avidin-biotin complex reagent (Vector Laboratories, Burlingame, CA, USA). Immunoreactive cells were then visualized by the addition of peroxidase substrates (3, 3-diaminobenzidine, or 3-amino-9-ethylcarbazole; Agilent) and counterstained with hematoxylin. Immunohistochemistry of p-H2A.X (Cell Signaling) was performed according to the EnVision^+^ Dual Link protocol (Agilent). Intracellular superoxide anion in renal sections was visualized using the sensitive fluorescent dye dihydroethidium (DHE; 2 μmol/L; Life Technologies, Carlsbad, CA, USA) followed by 4′,6-diamidino-2-phenylindole (DAPI) nuclear counterstain. All histological evaluations were conducted in a blinded fashion. Positive staining was quantified using Image Pro-Plus software (Media Cybernetics, Bethesda, MD, USA) and expressed as a percentage of the total area or number of positive cells (per glomerular cross section or per mm^2^).

### 2.4. Cell Culture

Human kidney 2 (HK2) proximal tubular cell line (ATCC Cat# CRL-2190, RRID:CVCL_0302; Manassas, VA, USA) was maintained in RPMI supplemented with 10% fetal bovine serum (FBS), 100 U/mL penicillin, 100 μg/mL streptomycin, 2 mM L-glutamine (Sigma-Aldrich, Saint Louis, MO, USA), 1% insulin–transferrin–selenium (Thermo Fisher Scientific) and 36 ng/mL hydrocortisone (Sigma-Aldrich). Cells were made quiescent by overnight incubation in serum-free RPMI and then pretreated for 90 min with hidrosmin (range of doses 0.1–1 mM) or vehicle of the highest dose (0.5% DMSO) before stimulation with either high-glucose (30 mM D-Glucose; Sigma-Aldrich) for 24 h, or a combination of human cytokines interleukin-6 (IL-6, 10^2^ U/mL) and interferon-γ (IFNγ, 10^3^ U/mL) (PeproTech, Rocky Hill, NJ, USA) for 6 and 24 h.

### 2.5. Cell Viability and Proliferation

Cell viability was measured by the 3-(4,5-dimethylthiazol-2-yl)-2,5-diphenyltetrazolium bromide tetrazolium (MTT) colorimetric assay (M-5655, Sigma-Aldrich). Briefly, HK2 cells were plated in 96-well plates (1.5 × 10^4^ cells/well) in RPMI with 10% FBS, synchronized overnight in serum-free medium, and then incubated in triplicate in medium containing hidrosmin, vehicle, 10% FBS (positive control), and 10% DMSO (negative control) for additional 24, 48 and 72 h. After treatment, the MTT solution (0.5 mg/mL) was added for 90 min, and the absorbance of the metabolized MTT was measured at λ = 570 nm in a plate reader. 

For cell proliferation assay at 24, 48, and 72 h, HK2 cells were seeded onto 96-well plates at densities of 2 × 10^4^, 1 × 10^4^, and 5 × 10^3^ cells/well, respectively, then synchronized overnight in serum-free medium and incubated in triplicate in RPMI with 10% FBS containing hidrosmin or vehicle, using 20% FBS and 10% DMSO as positive and negative controls, respectively. After treatment, the 5-bromo-2′-deoxyuridine (BrdU) solution was added during the last 2 h, and cells were processed according to the instructions of the BrdU Cell Proliferation ELISA Kit (ab126556, Abcam).

### 2.6. mRNA Expression Analysis

Total RNA from mouse tissues and cultured cells was extracted with TRIzol (Life Technologies). The resulting total RNA was quantified using a NanoDrop ND-1000 Spectrophotometer (NanoDrop Technologies, Wilmington, DE, USA). For each RNA sample, 1.5 μg of total RNA was reversely transcribed into cDNA using a High-Capacity cDNA Reverse Transcription Kit (Applied Biosystems, Foster City, CA, USA). Target gene expression was analyzed in duplicate by real-time PCR using a 7500 Fast Real-Time PCR System (Applied Biosystems) with TaqMan (Applied Biosystems) or SYBR Green Gene Expression (self-designed) detection assays (Supplementary Table S1). Expression levels of target genes were normalized to the *18S rRNA* housekeeping gene. The relative expression was determined using the formula 2^−∆Ct^.

### 2.7. Statistical Analysis

Results are shown as individual data and mean ± standard error of the mean (SEM) from separate animals and *n* = 3–6 independent culture experiments. Statistical analyses were performed using GraphPad Prism v.5 (GraphPad Software Inc., La Joya, CA, USA). Differences across groups were considered significant at *p* < 0.05 using either *t*-test and one or two-way ANOVA with post hoc Tukey or Bonferroni pairwise comparison test (when appropriate).

## 3. Results

### 3.1. In Vitro Renoprotective Effects of Hidrosmin

**Figure 1:**
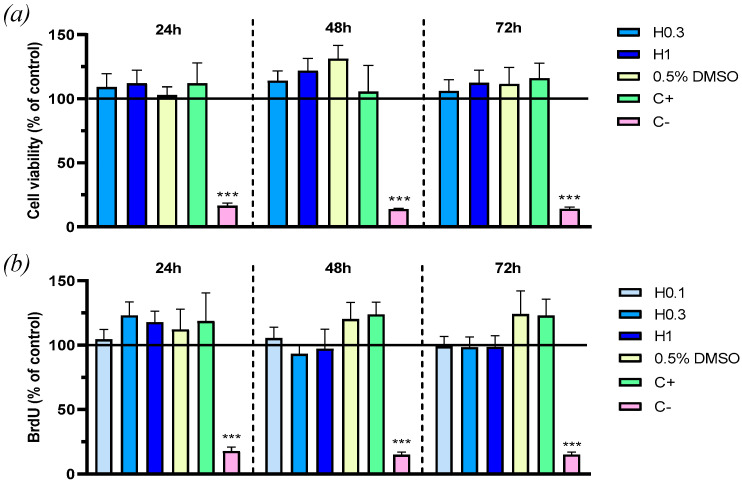
Hidrosmin-related effects on viability and cytotoxicity of HK2 cells: (**a**) MTT cell viability at 24, 48, and 72 h of incubation in medium with 0.5% FBS containing hidrosmin (H, 0.3 and 1 mM), vehicle of 1mM hidrosmin (0.5% DMSO), 20% FBS (positive control, C+), and 10% DMSO (negative control, C−); (**b**) BrdU cell proliferation assay at 24, 48, and 72 h incubation in medium with 10% FBS containing hidrosmin (H, 0.1, 0.3 and 1 mM), vehicle (0.5% DMSO), 20% FBS (C+) and 10% DMSO (C−). Bars represent the mean ± SEM of 6 experiments. *** *p* < 0.001 vs. basal (black line).

Before dealing with the in vivo experiments, we assessed the safety and functional effects of different concentrations of hidrosmin on human proximal tubule epithelial cells HK2. First, the MTT colorimetric assay demonstrated that neither hidrosmin nor its vehicle adversely affected cell viability of HK2 cells at 24, 48, or 72 h (Figure 1a). In addition, hidrosmin, at none of the doses tested (0.1, 0.3, and 1 mM), significantly reduced the cell growth, as determined by BrdU cell proliferation assay at 24, 48, and 72 h of incubation in medium with 10% FBS (Figure 1b). 

**Figure 2:**
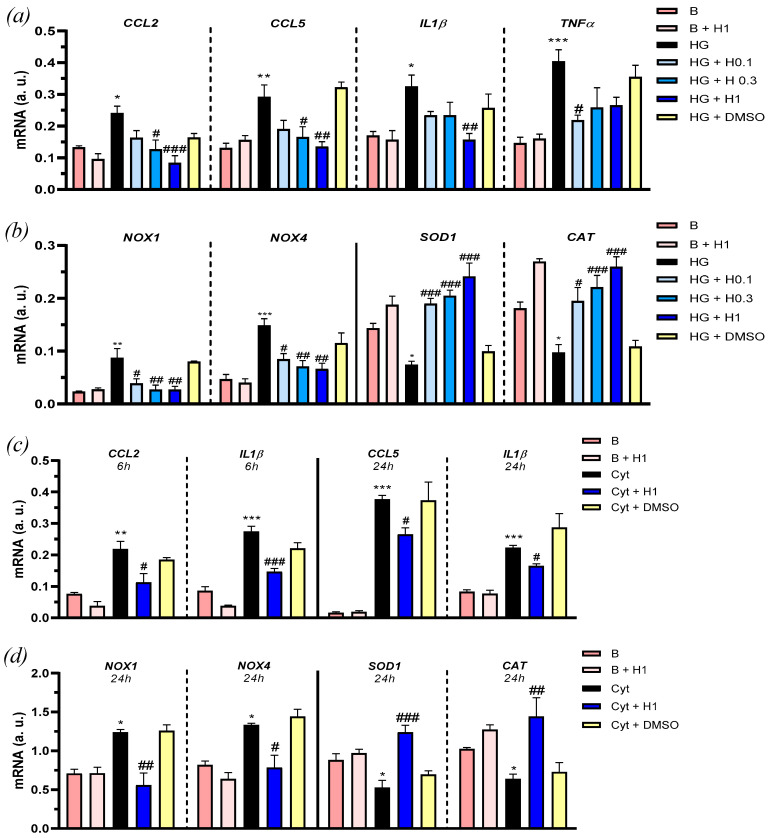
Effects of hidrosmin on inflammation and oxidative stress gene expression in HK2 cells. Real-time PCR analysis in HK2 cells exposed to medium with 0.5% FBS (B, basal) containing high-glucose (HG, 24h) (**a**,**b**) or cytokines (Cyt, 6 and 24 h) (**c**,**d**) in the absence/presence of hidrosmin (H0.1, H0.3 and H1, mM doses) or vehicle of its highest dose (0.5% DMSO). Bars represent the mean ± SEM of 3–4 experiments in duplicate. * *p* < 0.05, ** *p* < 0.01 and *** *p* < 0.001 vs. basal (B); # *p* < 0.05, ## *p* < 0.01 and ### *p* < 0.001 vs. stimulus (HG or Cyt).

The protective actions of hidrosmin were further evaluated in HK2 exposed to either high-glucose (30 mM D-glucose, 24 h) or a combination of inflammatory cytokines (IL-6 plus IFNγ, 6 and 24 h). Quantitative real-time PCR demonstrated that hidrosmin reduced, in a concentration-dependent manner, the gene expression of CC chemokines (*CCL2* and *CCL5*) and proinflammatory cytokines (*IL-1β* and tumor necrosis factor-α, *TNFα*) induced by 24 h high-glucose stimulation (Figure 2a). Similarly, hidrosmin pretreatment inhibited the gene expression profile in cells exposed to inflammatory cytokines (IL-6 plus IFNγ) (Figure 2c). Moreover, hidrosmin restored the expression of redox balance genes by preventing the prooxidant enzyme NADPH oxidase (*NOX1* and *NOX4* isoforms) and promoting the expression of antioxidant enzymes Superoxide dismutase-1 (*SOD1*) and Catalase (*CAT*) in HK2 cells exposed to high-glucose (Figure 2b), as well as to cytokines (Figure 2d).

### 3.2. Hidrosmin Treatment Ameliorates Renal Damage in Diabetic Mice

With the aim of testing whether hidrosmin can effectively affect the development of diabetic renal disease, we induced T1D by STZ injection in ApoE KO mice, a model of combined hyperglycemia and dyslipidemia that develops accelerated renal injury and resembles the morphology seen in DN patients [28]. Mice were randomized to hidrosmin treatment or control group, by daily intake of drinking water for 7 weeks. At the end of the study, we observed that hidrosmin treatment did not modify body weight, glycemia, and other biochemical parameters in diabetic mice, except for total cholesterol and low-density lipoprotein (LDL)-cholesterol, which presented a significant reduction when compared with the control group (Table 1).

**Figure 3:**
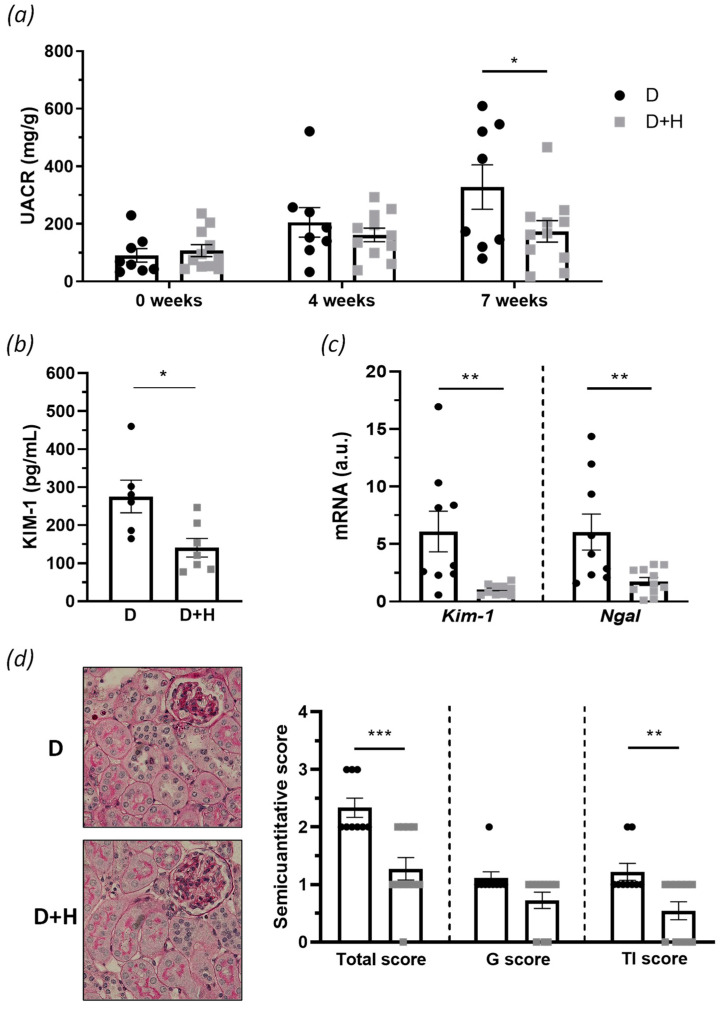
In vivo assessment of hidrosmin effect in experimental DN: (**a**) evolution of UACR levels in diabetic groups (control, D; hidrosmin-treated, D + H) along the study; (**b**) KIM-1 protein concentration in urine samples at final point; (**c**) real-time PCR analysis of *Kim-1* and *Ngal* in renal cortex. Values normalized by 18S are expressed in arbitrary units (a.u.); (**d**) representative images of PAS staining (magnification ×400) and assessment of glomerular (G), tubulointerstitial (TI), and total kidney semiquantitative score. Scatter dot plots represent individual values and the mean ± SEM of *n* = 6–9 animals (D group) and *n* = 7–11 animals (D + H group). * *p* < 0.05, ** *p* < 0.01, and *** *p* < 0.001 vs. D.

Diabetic mice exhibited progressive renal damage, as evinced by increased albuminuria levels over time (UACR; Figure 3a). Remarkably, hidrosmin administration ameliorated renal dysfunction by reducing UACR levels (% of decrease vs. control group: 47 ± 11, *p* = 0.0308; Figure 3a). The protective effect of hidrosmin was also confirmed by a significant decrease in the urinary KIM-1 levels, compared with untreated controls (% of decrease vs. control group: 49 ± 9, *p* = 0.0161; Figure 3b) and in the renal mRNA expression levels of *Kim-1* and neutrophil gelatinase-associated lipocalin (*Ngal*) (Figure 3c), which are hallmarks of kidney injury [29]. Histopathological analysis by PAS staining showed nephroprotective effects of hidrosmin, mainly ameliorating tubulointerstitial features in diabetic mice such as inflammatory infiltrate, tubular flattening, tubular atrophy, and interstitial fibrosis, which resulted in a significant reduction in tubulointerstitial and total kidney semiquantitative scores (Figure 3d). In addition, the glomerular changes were slightly affected, without observing significant appreciable changes.

### 3.3. Hidrosmin Treatment Attenuates Diabetes-Induced Renal Inflammation In Vivo

**Figure 4:**
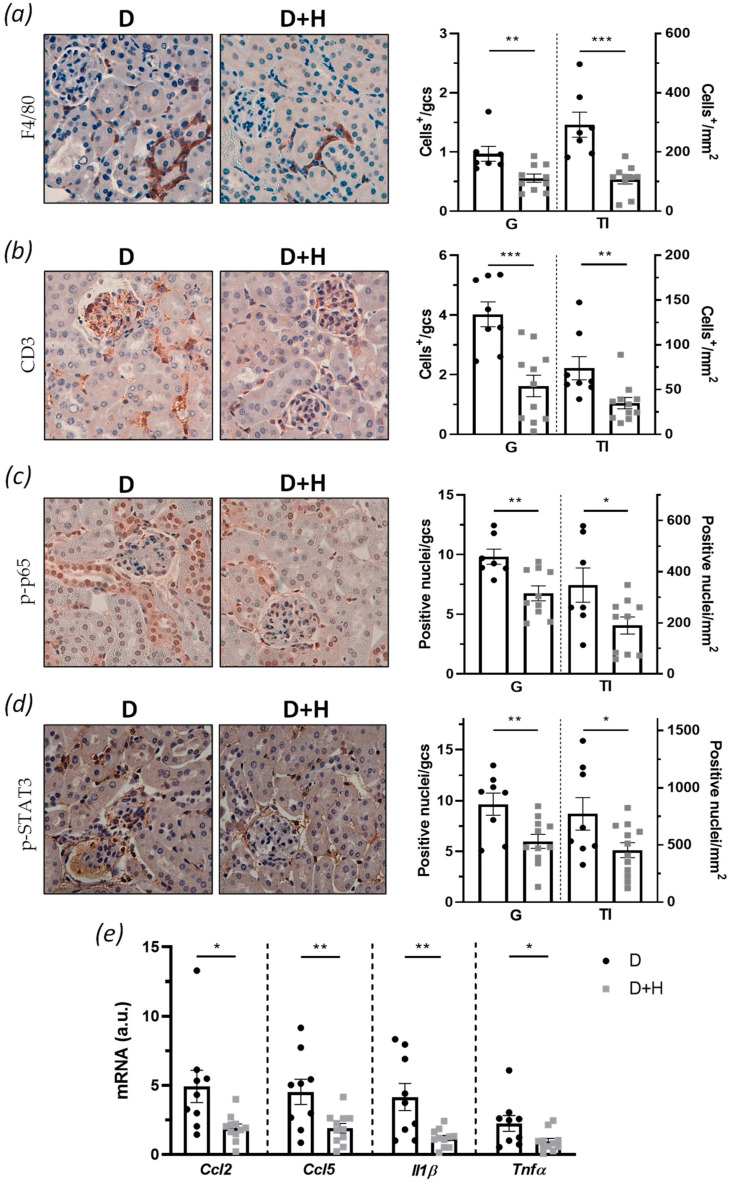
Anti-inflammatory effect of hidrosmin in diabetic kidney: (**a**–**d**) representative images (original magnification, ×400) and quantification of positive immunostaining for (**a**) CD3, (**b**) F4/80, (**c**) p-p65, and (**d**) p-STAT3 in diabetic groups (control, D; hidrosmin, D + H). Values are expressed as positive cells per glomerular cross section (gcs) in glomeruli (G) or mm^2^ in tubulointerstitium (TI); (**e**) real-time PCR analysis of inflammatory genes in renal cortex. Values normalized to 18S are expressed as arbitrary units (a.u.). The graphs represent individual values and mean ± SEM of *n* = 7–9 animals (D group) and *n* = 10–11 animals (D + H group). * *p* < 0.05, ** *p* < 0.01, and *** *p* < 0.001 vs. D.

We investigated whether hidrosmin treatment affects inflammatory pathways involved in diabetic kidney damage and the renal levels of leukocyte and proinflammatory markers, by immunohistochemistry and real-time PCR. Compared with untreated control mice, hidrosmin-treated mice displayed a lower number of infiltrating CD3^+^ T lymphocytes (Figure 4a) and F4/80^+^ macrophages (Figure 4b), both in the glomerular and tubulointerstitial compartments. Hidrosmin administration also prevented the activation of NF-κB and JAK/STAT, two important inflammatory signaling pathways in DN, as demonstrated by significantly reduced levels of phosphorylated transcription factors p-p65 (Figure 4c) and p-STAT3 (Figure 4d) in renal sections. Moreover, in correlation with in vitro results, hidrosmin significantly downregulated the gene expression of inflammatory chemokines and cytokines (*Ccl2*, *Ccl5*, *Il1β,* and *Tnfα*) in diabetic kidneys (Figure 4e).

### 3.4. Hidrosmin Reduces Oxidative Stress and Senescence Markers in Diabetic Kidney

**Figure 5:**
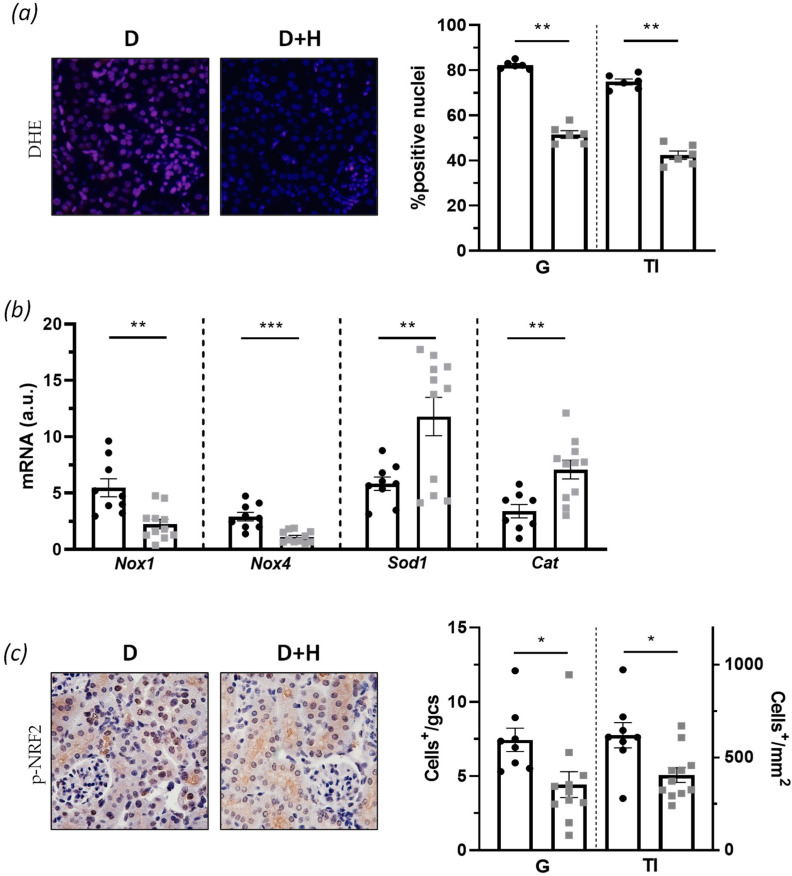
Antioxidant effect of hidrosmin in the diabetic kidney: (**a**) representative images of kidney sections stained with DHE in red, and DAPI in blue (merged images; magnification x400) and quantification of positive cells in renal sections from untreated control (D) and hidrosmin-treated (D + H) mice; (**b**) real-time PCR analysis of prooxidant (*Nox1* and *Nox4*) and antioxidant (*Sod1* and *Cat*) genes. Values normalized to 18S are expressed as arbitrary units (a.u.); (**c**) representative images (magnification ×400) of p-NRF2 immunodetection and quantification of positive cells in glomeruli (G) and tubulointerstitium (TI) of diabetic mice. Bars represent individual values and the mean ± SEM of *n* = 8–9 animals (D group) and *n* = 11 animals (D + H group). * *p* < 0.05, ** *p* < 0.01, and *** *p* < 0.001 vs. D.

We next examined the impact of hidrosmin treatment on the renal levels of oxidative stress markers in diabetic mice. First, superoxide anion detection by the sensitive fluorescent probe DHE demonstrated that hidrosmin-treated mice produced lower ROS levels when compared with the untreated control group (Figure 5a). Furthermore, hidrosmin significantly reduced the gene expression of *Nox1* and *Nox4* prooxidant enzymes in diabetic kidneys while increasing antioxidant genes *Sod1* and *Cat* by approximately a twofold increase versus the untreated group (Figure 5b). Finally, immunohistochemistry of p-NRF2 showed a decreased activation of the antioxidant pathway both in glomeruli and tubulointerstitium (Figure 5c).

**Figure 6:**
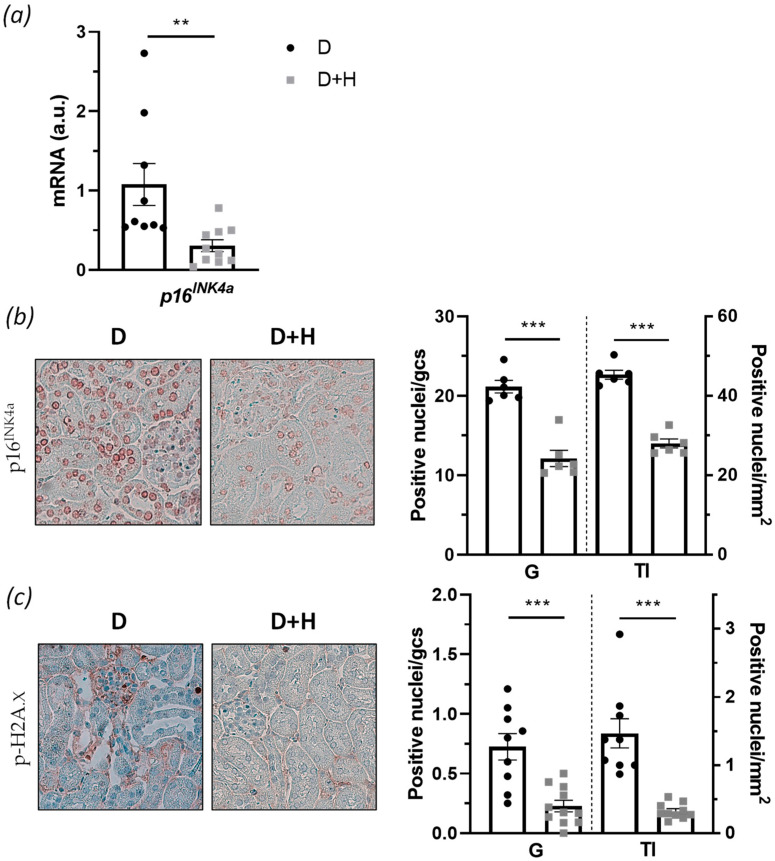
Hidrosmin effects on senescence markers in diabetic kidneys: (**a**) real-time PCR analysis of the cell cycle inhibitor p16^INK4a^ gene expression in renal samples. Values normalized to 18S gene are expressed as arbitrary units (a.u.); (**b**,**c**) representative images (magnification ×400) and quantification of p16^INK4a^ protein (**b**) and DNA damage biomarker p-H2A.X (**c**) in kidney sections from of untreated (D) and hidrosmin-treated (D + H) diabetic mice. The graphs represent individual values and the mean ± SEM of *n* = 9 animals (D group) and *n* = 11 animals (D + H group). ** *p* < 0.01 and *** *p* < 0.001 vs. D.

Considering the several improvements observed in hidrosmin treatment and given that diabetes accelerates renal cell senescence [30], we further analyzed the effect of hidrosmin on senescence markers in diabetic mouse kidneys. First, we studied the expression of cyclin-dependent kinase inhibitor p16^INK4a^, a key cell cycle factor highly involved in the senescence environment [31]. By real-time PCR analysis, we found a significant 71 ± 7% reduction in *p16 ^INK4a^* gene expression in the hidrosmin-treated group, compared with the untreated control group (Figure 6a). Moreover, immunohistochemistry showed a decreased expression of p16^INK4a^ protein, both in glomeruli and tubulointerstitium (% decrease vs. control: 43 ± 1% and 38 ± 1%, respectively) (Figure 6b). Finally, with the aim of studying kidney DNA damage in diabetic mice, we carried out immunohistochemistry of p-H2A.X, due to the fact that phosphorylation of this histone is an early response to DNA damage and a marker of aging [32]. The results unveiled that hidrosmin markedly decreased the number of p-H2A.X positive nuclei in glomerular and tubulointerstitial compartments (Figure 6c), therefore indicating the antisenescence effect of the synthetic flavonoid.

## 4. Discussion

DM cases are expected to increase to 642 million by 2040, and considering the current outlook of this illness, the predicted augmentation for DN is in a similar way. In addition to the huge health expenditure dedicated to DM and its chronic complications, patients with diabetic kidney disease still have more probability of suffering cardiovascular diseases, infections, cancers, etc. [4]. Despite the enormous advances on cardiovascular and renal protection provided by the SGLT2i and GLP-1 RA introduced in clinical practice for diabetic patients [15,16,17], no drug targeting inflammation and redox balance signaling pathways, key elements in the genesis and progression of DN [7,18], is available so far. Flavonoid compounds seem to be a promising alternative to treat chronic DM complications due to their outstanding pharmacological effects including anti-inflammatory, antioxidant, antidiabetic, and antihypertensive, as evinced in experimental models and clinical studies [20,33,34]. The synthetic flavonoid hidrosmin, a diosmin-derived flavone with vasoprotective effects [35], is a venotonic agent used for many years in patients suffering from chronic venous insufficiency mainly due to its antiedematous properties and other beneficial effects (i.e., in swelling and leg cramps), more effective in some cases than diosmin [21,36]; however, the therapeutic effects of hidrosmin in other chronic diseases remains to be explored. This study is the first preclinical evaluation of the effects and underlying mechanisms of hidrosmin therapy in diabetic-induced kidney disease.

First, the in vitro study in human tubular cells demonstrated that hidrosmin did not exhibit any cytotoxic or antiproliferative effects at the concentrations and time points investigated, thus confirming the safety and biocompatibility similar to other flavonoid compounds [37]. Further, hidrosmin treatment downregulated the cytokine expression in renal cells exposed to high-glucose and/or inflammatory conditions and also modulated redox balance genes. These findings prompted us to investigate in vivo the hidrosmin activity in an experimental mouse model of DN.

The STZ-induced T1D in ApoE KO mice is a model of combined hyperglycemia and hyperlipidemia widely used for studying pathogenic mechanisms and nephroprotective therapies in DN because of similarities with human disease. These include elevated UACR, renal pathological changes (glomerular hypertrophy, glomerular basement membrane thickening, mild/moderate mesangial matrix expansion, tubule enlargement, and tubular cell atrophy), and increased oxidative stress and inflammatory markers [28,38,39]. In this work, oral administration of hidrosmin to overtly diabetic mice did not modify the metabolic and biochemical parameters except for a significant decrease in total and LDL cholesterol. Studies in patients and animal models have demonstrated that DM aggravates dyslipidemia and cardiovascular risk and that hyperlipidemia facilitates DN development, perpetuating a cycle of metabolic disorders [39,40,41]. In T1D and T2D patients, lipid control appears to be important in the prevention and treatment of macro- and microvascular complications, including DN [42,43]. Previous reports have described the antihyperlipidemic potential of different flavonoids in experimental models of cardiovascular disease [44,45]. Likewise, we suggest that hidrosmin could be tested as coadjutant therapy with the current lipid-lowering drugs to reduce cardiovascular risk and delay the progression of DN.

Diabetes-derived renal damage is usually evaluated by systemic parameters such as UACR [46] and protein renal markers, including KIM-1 and Ngal [29]. These markers became ameliorated in this investigation after hidrosmin treatment, without any observable toxicity. Similar effects have been observed with silymarin [47] and green tea extract [48] in diabetic patients. Furthermore, we observed that hidrosmin ameliorated the renal histological changes in diabetic mice, including mesangial expansion, tubular atrophy and inflammatory infiltrate, giving the flavonoid important chances to alleviate renal dysfunction.

The immunoinflammatory response is crucial in the development and progression of DN, and this process is mediated by a burden of immune system elements such as lymphocytes, macrophages, and their released mediators [49]. Indeed, upregulation of proinflammatory cytokines and chemokines in the diabetic environment can promote leukocyte recruitment and affect renal cell functions via receptor-mediated processes, further contributing to renal damage [6]. The interaction between chemokines and their receptors leads to the recruitment of inflammatory cells into the damaged kidney, and several investigations revealed that CCL2 and CCL5 chemokines have important roles in the DN pathogenesis [8,50,51]. Moreover, besides chemokines, proinflammatory cytokines such as TNFα, IFNγ, IL-1 and IL-6 contribute to the development and maintenance of inflammation [7,52]. Our work reveals a lower renal content of T cells and macrophages in hidrosmin-treated mice, and a concomitant downregulation of chemokines (CCL2 and CCL5) and cytokines (TNFα and IL-1). These findings reinforce the anti-inflammatory action of hidrosmin in the diabetic kidney, which is in line with the reported action of different flavonoids in different experimental models [47,53,54]. 

In addition to the improvement in renal inflammation upon hidrosmin treatment, we also detected a decreased activity of NF-κB and JAK/STAT, two signaling pathways that control key cellular responses during the onset and progression of DN [7,49]. Dysregulated activation/expression of NF-κB, JAK/STAT, and their target genes have been detected in kidney biopsies from diabetic patients [8,55]. In experimental models, either gene deficiency or pharmacological inactivation of different family members (e.g., JAK2, STAT1, and STAT3) further implicates both pathways in DM and its chronic complications [56,57]. This study indicates that the renoprotective effects of hidrosmin in diabetic kidneys are mediated by the concerted inhibition of NF-κBp65 and STAT3 transcription factors, although further studies are needed to identify the exact mechanism of the compound. Our findings are consistent with previous evidence showing that different flavonoids relieve renal inflammation in the diabetic milieu by inhibiting NF-κB [23,53,58] and STAT signaling [59,60].

Chronic hyperglycemia enhances inflammation, promotes ROS generation, and hampers antioxidant systems thus contributing to oxidative stress and renal injury [18,61]. In the kidney, NADPH oxidase-derived ROS regulates gene expression, renal blood flow, and cell responses through different mechanisms including activation of kinases, transcription factors, and the inflammasome [7]. Accordingly, our study demonstrates that hidrosmin decreases superoxide anion production in diabetic kidneys and also suppresses the gene expression levels of NOX1 and NOX4, which are relevant ROS-generating enzymes contributing to DN. Indeed, pharmacologic or genetic targeting of NOX1/4 has been shown to ameliorate proteinuria and renal damage in diabetic mice [62,63,64]. Our findings are in line with previous studies showing reduced NOX1 enzyme by the flavone cardamonin in cisplatin-induced nephrotoxicity model [65], and the involvement of NOX4 on the nephroprotective actions of the flavanone glycoside naringin in diabetic rats [66]. Remarkably, unlike these natural flavonoids, hidrosmin displays a dual effect on both NOX1 and NOX4, similar to the results reported with the small synthetic molecule GKT137831, a selective NOX1/NOX4 inhibitor [67]. 

Furthermore, the antioxidant capacity of the kidneys was improved with the hidrosmin treatment, demonstrated by an increase in the expression of SOD1 and CAT, honoring the classical antioxidant capacity of flavonoids described in the literature [53,54,58]. Nevertheless, we found a downregulation of the NRF2 pathway activation after the treatment, a fact we can attribute to hidrosmin and its clear inhibition of the NF-κB pathway, which controls inflammation and ROS generation and influences NRF2 [68]. Under these conditions, NRF2 pathway activation could be less necessary, which therefore does not exclude the possible activation during disease onset or early symptomatic stages.

Oxidative stress and inflammation potentiate each other and are major inducers of an accelerated kidney aging process characterized by the increased levels of p16^INK4a^, DNA damage markers, etc. [14,69]. Hyperglycemic conditions stimulate the expression of cyclin kinase inhibitor p16^INK4a^ and accelerate renal cell senescence [70]. Moreover, p16^INK4a^ levels correlate directly with the degree of glomerular lesions and tubule atrophy in T2D patients [71]. Our study reveals that hidrosmin prevents the upregulated expression of p16^INK4a^ in diabetic kidneys, indicating a deceleration of diabetes-induced premature senescence similar to the effect described for the dietary flavonoid quercetin in diet-induced obesity models [72]. Concomitantly, hidrosmin ameliorates the phosphorylation of H2A.X histone, a marker of DNA double-strand breaks and the first step in recruiting and localizing DNA repair proteins [73,74]. It has been reported that NOX1- and NOX4-generated ROS promote senescence by activating the p53 and p16^INK4a^ pathways [75], whereas *Nox1* deficiency attenuates H2AX phosphorylation and premature senescence in early stage DN [76]. Identification of senescent phenotype pathways that could be directly or indirectly affected by hidrosmin will help understand the potential senolytic activity of hidrosmin in renal cells. 

The limitations of this study could be reflected in three main points: (1) The results of this study have been obtained in the streptozotocin ApoE KO model, which combines a T1D-like model with marked hyperlipidemia, due to a genetic deficiency in ApoE. Although patients with both T1D and T2D can have lipid abnormalities, they usually do not show intense dyslipidemia seen in this experimental model. (2) The study was mainly designed to find out the effect of hidrosmin on the early stages of DN. (3) Although diabetic kidney disease in T1D and T2D share a number of common pathogenetic mechanisms, taking into account the tremendous prevalence of T2D, the potential beneficial effects of hidrosmin in T2D mouse models should be explored.

In conclusion, this work demonstrates that hidrosmin affects various signaling pathways and ameliorates experimental DN through the inhibition of crucial pathogenic processes such as inflammation, oxidative stress, and accelerated senescence. Our results support hidrosmin as a candidate to improve the diabetic kidney disease symptomatology, alleviate renal dysfunction, and delay renal injury. Nevertheless, future efforts are needed to optimize hidrosmin usage and applications in diabetic patients as a coadjuvant therapy. 

## Figures and Tables

**Table 1:** Effect of hidrosmin on metabolic parameters in diabetic mice. Abbreviations: KBWR, kidney–body weight ratio; GLU, glucose; BUN, blood urea nitrogen; ASAT, aspartate aminotransferase; ALAT, alanine aminotransferase; TC, total cholesterol; TG, triglycerides; HDL-C, high-density lipoprotein cholesterol; LDL-C, low-density lipoprotein cholesterol. Values are mean ± SEM of the total number of animals per group (*n* = 9–11). * *p* < 0.05 and ** *p* < 0.01 vs. diabetic group.

Metabolic Parameters	Diabetic	Diabetic + Hidrosmin
Body Weight (g)	22.1 ± 0.7	21.9 ± 0.8
KBWR (g/g)	9.8 ± 0.7	9 ± 0.4
GLU (mg/dL)	484.8 ± 22.7	420.3 ± 41,4
Water intake (mL/day)	30 ± 3.4	28.9 ± 3.1
Urea (mg/dL)	70.1 ± 6.8	64.5 ± 4.2
BUN (mg/dL)	32.8 ± 3.2	30.1 ± 2
ASAT (UI/L)	212 ± 28.8	197.1 ± 60.1
ALAT (UI/L)	79.1 ± 13.7	93.8 ± 15.5
TC (mg/dL)	911.7 ± 74.5	573.3 ± 83.9 **
TG (mg/dL)	228.7 ± 47	140.6 ± 21.2
HDL-C (mg/dL)	92.2 ± 6.7	79.4 ± 5.1
LDL-C (mg/dL)	750.9 ± 61.6	513.1 ± 71.2 *

## Data Availability

Data is contained within the article and Supplementary Material.

## Supplementary Materials

The following are available online at https://www.mdpi.com/article/10.3390/antiox10121920/s1, Figure S1: Chemical structure of hidrosmin; Table S1: Human and mouse primers used for real-time PCR.
