# Supplementary material for: Nephroprotective Effects of Synthetic Flavonoid Hidrosmin in Experimental Diabetic Nephropathy
Source: Antioxidants (Basel). 2021 Nov 29;10(12):1920. doi: 10.3390/antiox10121920 (PMC8750193; doi:10.3390/antiox10121920)
Supplement: Supplementary file 1 [file antioxidants-10-01920-s001.zip › antioxidants-1453674-supplementary.pdf]

Gene		Taqman assay ID	
**Name**	**ID**	**Human**	**Mouse**
CCL2/Ccl2	6347/20296	Hs00234140_m1	Mm00441242_m1
CCL5/Ccl5	6352/20304	Hs00174575_m1	Mm01302428_m1
IL1-β/Il1-β	3553/16176	Hs01555410_m1	Mm00434228_m1
TNFα/Tnfα	7124/21926	Hs00174128_m1	Mm00443258_m1
p16^{INK4a}	12578	-	Mm00494449_m1
Ngal	16819	-	Mm01324470_m1
Kim-1	171283	-	Mm00506686_m1
SOD1/Sod1	6647/20655	Hs00533490_m1	Mm01344233_g1
CAT/Cat	847/12359	Hs00156308_m1	Mm00437992_m1
18s rRNA	X03205.1	4310893E	4310893E
**Gene**		**SYBR Green Primers**	
**Name**	**ID**	**Forward**	**Reverse**
NOX1	27035	TAAAGGCTCACAGACCCTGC	GAGCCCTTCTAGGCAACAGG
Nox1	237038	CCAACAGGCCATGGATGGAT	CACTCCAGTAAGCCAGCAA
NOX4	50507	CACCAGATGTTGGGGCTAGG	TGATCCTCGGAGGTAAGCCA
Nox4	50490	CCCTCCTGGCTGCATTAGTC	AACCCTCGAGGCAAAGATCC
18S rRNA	X03205.1	CCGTCGTAGTTCCGACCATAA	CAGCTTTGCAACCATACTCCC

Supplementary Table S1. Human and mouse primers used for real-time PCR.

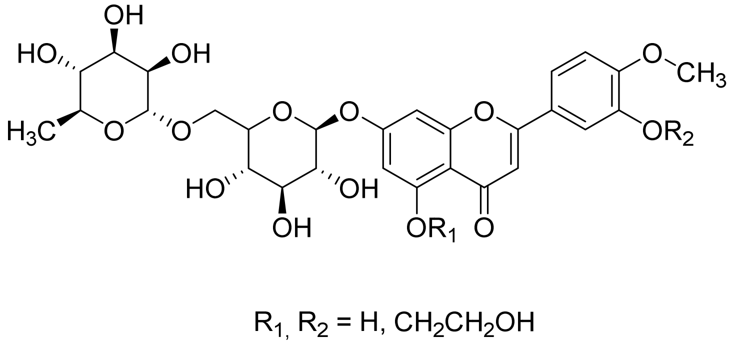

Supplementary Figure S1. Chemical structure of hidrosmin.
